# Feasibility and Policy Implications of a Pragmatically Adapted Pediatric-Inspired Induction Regimen for Adults with Acute Lymphoblastic Leukemia in a Resource-Restricted Setting: A Prospective Observational Study

**DOI:** 10.3390/healthcare14081038

**Published:** 2026-04-14

**Authors:** Sadia Qazi, Hafsa Fayyaz, Bilal Ahmad, Abdal Ahmad, Syeda Sama Bilal, Aiman Ajmeer, Humna Aziz

**Affiliations:** 1 Department of Anatomy, College of Medicine, Alfaisal University, Riyadh 11533, Saudi Arabia; 2 Department of Oncology, Pakistan Institute of Medical Sciences, Islamabad 04485, Pakistan; drhafsahamid@yahoo.com; 3 Department of Oncology, Kuwait Teaching Hospital, Peshawar 25140, Pakistan; bilalwazirkmc@gmail.com (B.A.); s.samabilal@gmail.com (S.S.B.); 4 Department of Medicine, Mercy Teaching Hospital, Peshawar 25150, Pakistan; abdalmomand@gmail.com (A.A.); humniazizgul@gmail.com (H.A.); 5 Department of Pathology, Peshawar Medical College, Riphah International University, Peshawar Campus, Peshawar 25160, Pakistan; aimanajmeer15th@gmail.com

**Keywords:** acute lymphoblastic leukemia, pediatric-inspired induction, LMICs, feasibility, diagnostic access, affordability, Pakistan, health policy

## Abstract

**Highlights:**

**What are the main findings?**

- In 200 adults with Philadelphia chromosome-negative Acute lymphoblastic leukemia [(Ph(−)ALL] treated in a Pakistani public hospital, an adapted pediatric-inspired induction pathway achieved an 83.0% complete remission rate with low early mortality (2.0%) and treatment abandonment (1.5%) rates.
- Delivery was feasible in a resource-constrained setting, but guideline-complete diagnostics remained financially inaccessible despite chemotherapy subsidy.

**What are the implications of the main findings?**

- Pediatric-inspired induction can be implemented in LMIC public sector oncology through pragmatic adaptation and coordinated service packaging.
- The main system barriers are diagnostic financing and the reliable procurement of critical agents, making these the most actionable targets for policy reform.

**Abstract:**

**Background**: Acute lymphoblastic leukemia (ALL) requires intensive induction, but implementation of pediatric-inspired regimens in low- and middle-income countries is constrained by diagnostic gaps, procurement instability, and limited supportive-care capacity. We evaluated the feasibility, safety, and affordability of a pragmatically adapted pediatric-inspired induction regimen for adults with Philadelphia chromosome-negative Ph(−) ALL in a Pakistani tertiary hospital. **Methods**: In this prospective single-center cohort study at the Pakistan Institute of Medical Sciences (December 2024–June 2025), consecutive adults aged 18–50 years with newly diagnosed Ph(−)ALL received an adapted pediatric-inspired induction regimen. The primary outcome was complete remission (CR) after induction, with or without extended induction. Secondary outcomes were early mortality, treatment abandonment, grade 3–4 toxicities, and service delivery feasibility indicators. Affordability was assessed against household income. **Results**: Among 200 adults (mean age 30.3 ± 8.8 years; 65.5% male), 39.5% presented with WBC ≥ 30 × 10^9^/L and 88.0% with platelets < 50 × 10^3^/µL. CR was achieved in 83.0% of patients. Early mortality was 2.0%, and treatment abandonment was 1.5%. Grade 3–4 toxicities included febrile neutropenia (15.0%) and sepsis (7.5%). The Day-30 evaluability was high (96.5%). Observed out-of-pocket diagnostic costs were USD 119, whereas a guideline-complete diagnostic package would cost USD 929, equivalent to 3–6 months of income for households in the poorest quintile. **Conclusions**: This adapted pediatric-inspired induction regimen was operationally deliverable in a resource-restricted hospital and produced favorable induction-phase outcomes. Limited diagnostic capacity and a lack of financial protection for testing remain barriers to risk-adapted care. Expanding subsidies for essential diagnostics and stabilizing the procurement of critical agents may yield the greatest implementation gains.

## 1. Introduction

Acute lymphoblastic leukemia (ALL) is an aggressive hematologic malignancy that requires rapid diagnosis and intensive therapy to improve outcomes [1]. Effective ALL care functions as a time-sensitive care bundle that depends on chemotherapy selection, diagnostic capacity, drug procurement, quality assurance, and financial protection. In high-income countries (HICs), multi-agent pediatric-inspired regimens incorporating asparaginase and high-dose methotrexate have significantly improved remission and long-term survival in fit adults [2,3,4]. These approaches rely on advanced diagnostics, including flow cytometric minimal residual disease (MRD) monitoring, conventional cytogenetics, and genetic profiling, as well as comprehensive supportive care [5,6]. They also enable the integration of targeted therapies, such as tyrosine kinase inhibitors, for Ph-like ALL [7]. Therefore, outcomes are closely linked to health system readiness, not solely to regimen design.

In many low- and middle-income countries (LMICs), adults with ALL often present with a higher disease burden and limited diagnostic stratification [8,9]. Delays in diagnosis and major financial barriers frequently lead to the empirical use of less intensive regimens [8,9,10]. Without routine cytogenetic and molecular profiling, risk-adapted therapy is rarely possible, although adult outcomes can approach pediatric benchmarks when precise risk stratification and MRD-guided decisions are implemented [3,4]. In resource-constrained settings, clinicians often pragmatically adapt protocols rather than reproducing them wholesale [11,12,13]. This raises an important question: which elements of pediatric-inspired induction can be reliably delivered in public-sector systems, and which failures in diagnostics, supply chains, or supportive care translate into avoidable toxicity, abandonment, or early mortality?

Pakistan exemplifies these constraints. The country lacks a unified national cancer registry, and health spending relies heavily on out-of-pocket payments [9,10,14]. Structural factors, including high rural residence, limited health literacy, and delayed engagement with formal cancer services, contribute to late presentation [15,16]. Public facilities, such as the Pakistan Institute of Medical Sciences (PIMS) in Islamabad, function as safety-net facilities but face chronic overcrowding and staffing constraints [17]. Diagnostic assessment is commonly limited to morphology and immunophenotyping, while cytogenetics, MRD testing, and Ph-like molecular profiling are largely unavailable because of cost and lack of subsidy coverage [9,10]. Drug supply volatility, particularly for asparaginase, alongside shortages of blood products and limited ICU capacity, further compromises timely supportive care [13,18,19,20]. Therefore, this study evaluated the feasibility, early safety, and affordability of a pediatric-inspired induction regimen for adult Ph(−)ALL at PIMS, with the objectives of assessing diagnostic feasibility, therapeutic pathway completion, early clinical outcomes, and economic burden relative to household income.

## 2. Materials and Methods

### 2.1. Study Design and Ethical Approval

This prospective, single-center, observational cohort study assessed the feasibility, safety, and induction-phase outcomes of a pragmatically adapted, pediatric-inspired regimen in adults with Ph(−)ALL. The study was designed as an implementation-focused evaluation of routine public-sector oncology care, with an emphasis on service delivery, specifically diagnostic access, treatment completion, toxicity management, and affordability rather than comparative efficacy. Care was delivered as part of routine clinical practice; there was no randomization, masking, or experimental allocation involved. A randomized design was considered impractical owing to diagnostic constraints, financial barriers, and the absence of a suitable comparator regimen in the local setting of this study. Reporting followed the STROBE guidelines, where applicable, for observational cohorts [21]. This study was approved by the Institutional Ethics Review Committee of the Pakistan Institute of Medical Sciences (PIMS), Islamabad (Reference: F-5-2/2024(ERRC)/PIMS). All participants provided written informed consent prior to their enrollment. This study was conducted in accordance with the Declaration of Helsinki.

### 2.2. Study Setting

The study was conducted at the Department of Medical Oncology, Pakistan Institute of Medical Sciences (PIMS), Islamabad, Pakistan, between December 2024 and June 2025. PIMS is a 1300-bed tertiary teaching hospital and referral center serving the Islamabad Capital Territory and the adjacent provinces of Punjab and Khyber Pakhtunkhwa, functioning as a public-sector safety-net facility for advanced presentations. The oncology service comprises 18 inpatient beds (nine male and nine female) and an outpatient chemotherapy bay staffed by dedicated oncology nurses. Given the study volume relative to fixed ward capacity, a hybrid inpatient-outpatient model was used: inpatient beds were prioritized for treatment initiation and complication management, while clinically stable phases of induction were managed in the outpatient chemotherapy bay. Care is provided by faculty physicians, rotating medical officers, postgraduate residents, and house officers with continuous 24 h coverage. Morphological review and immunophenotyping were performed by four laboratory-based pathologists; there were no dedicated hematologists. Regular academic activities included daily teaching rounds, twice-weekly multidisciplinary meetings with critical care, pulmonology, and dermatology, and rotating grand rounds. This institutional context is documented to support the interpretation of feasibility outcomes as health service deliverables.

Induction chemotherapy costs for enrolled patients were covered under the Sehat Sahulat Program (commonly referred to as the Sehat Card), a government health insurance scheme providing fully subsidized inpatient care including secondary and tertiary oncology services at accredited public and private hospitals, with annual family coverage of up to PKR 1,000,000. The program originated in Khyber Pakhtunkhwa in 2015–2016 and has since expanded to Punjab, the Islamabad Capital Territory, and other regions [22,23]. Sehat Card coverage does not extend to advanced diagnostics, including conventional cytogenetics, MRD testing using flow cytometry, or Ph-like molecular profiling. Accordingly, the observed patient out-of-pocket expenditure reflected only unsubsidized costs for BCR-ABL1 RT-PCR, immunophenotyping, and procedural fees.

### 2.3. Participants

Adults aged 18–50 years with newly diagnosed Ph(−)ALL were included. Additional inclusion criteria were an Eastern Cooperative Oncology Group (ECOG) performance status of 0–2, adequate organ function, defined as a serum creatinine level below 2.0 mg/dL and total bilirubin below 2.0 mg/dL, and brief pre-referral corticosteroid exposure, reflecting real-world referral patterns in low- and middle-income countries.

#### 2.3.1. Exclusion Criteria

Patients were excluded based on the following criteria assessed prior to starting induction: BCR-ABL1 positivity (*n* = 27), Burkitt leukemia/lymphoma (L3 morphology) *(n* = 11), pregnancy *(n* = 3), uncontrolled infection (*n* = 6), recent malignancy requiring active treatment (*n* = 2), and CNS involvement necessitating a modified therapeutic approach (*n* = 1).

#### 2.3.2. Patient Screening and Enrollment

A total of 250 patients were screened between December 2024 and April 2025. Because BCR-ABL1 testing carries a median turnaround time of 6 days, defined as the interval from sample submission to availability of the final report in the clinical record, induction was initiated empirically prior to the return of Ph-status results. This pragmatic decision prevented treatment delays while awaiting a diagnostic confirmation. After applying the exclusion criteria, 200 patients were enrolled and initiated on the adapted pediatric-inspired induction regimen. The extended study period through June 2025 reflects the window of active clinical follow-up and data collection following enrollment, which closed in April 2025.

### 2.4. BCR-ABL1 Testing

All patients underwent qualitative reverse transcription polymerase chain reaction (RT-PCR) for BCR-ABL1 (p190/p210 isoforms) testing. Testing was performed either in the institutional molecular laboratory or via external accredited laboratories, with a median turnaround time of six days. This assay was prioritized because BCR-ABL1 positivity requires prompt addition of tyrosine kinase inhibitor therapy, and delayed confirmation risks suboptimal treatment in Ph-positive cases [7,24]. The use of in-house versus outsourced testing was documented as a service-delivery characteristic reflecting institutional throughput capacity rather than a biological variable; longer outsourced turnaround times may delay the initiation of targeted therapy in such cases.

### 2.5. Diagnostic Workflow

Baseline assessment comprised complete blood count, serum chemistry, lactate dehydrogenase, and bone marrow aspiration with a morphological examination. Immunophenotyping was performed using available antibody panels to distinguish between B-ALL and T-ALL. In cases where lineage could not be assigned due to insufficient data, including sample degradation or incomplete antibody panels with the unavailability of cCD79a, cCD3, and MPO, the case was designated “Immunophenotype not classifiable (technical limitations)” to distinguish resource-driven diagnostic gaps from true ambiguous-lineage leukemia [25]. Diagnostic feasibility was conceptualized as “access achieved” (test obtained and resulted) rather than “test indicated,” because affordability and availability are the primary determinants of test completion in this setting.

### 2.6. Diagnostics Not Routinely Available

Conventional cytogenetics, flow cytometry-based measurable residual disease (MRD) assessment, and molecular profiling for Ph-like ALL were not performed in any of the 200 enrolled patients (0/200, 0%). These investigations are not covered under the Sehat Card program or any other provincial health scheme at this facility; coverage extends to induction chemotherapy but explicitly excludes the molecular diagnostics. The tests are available only at a small number of major urban referral centers and remain unaffordable for the patient population served by the PIMS. Accordingly, risk stratification was limited to morphology and immunophenotyping, and MRD-guided intensification or de-escalation was not feasible in routine care settings. These constraints mirror the structural diagnostic gaps documented across LMIC oncology settings [26,27,28,29].

### 2.7. Treatment Protocol in Routine Care

Patients received a pragmatically adapted, pediatric-inspired regimen selected to match local drug availability, monitoring capacity, and staffing resources. The treatment approach was based on CALGB 10403 [30], while being operationalized within the limits of routine public-sector care. In practical terms, the elements most reliably deliverable in this setting were the core induction chemotherapy backbone, scheduled central nervous system (CNS) prophylaxis with intrathecal therapy, and standardized day-30 bone marrow response assessment. Thus, the principal pediatric-inspired induction components preserved in routine care were the anthracycline-vincristine-corticosteroid-asparaginase backbone, scheduled intrathecal CNS prophylaxis, and protocolized early marrow reassessment to guide subsequent management.

Pragmatic adaptation in our setting did not involve abandonment of this pediatric-inspired induction strategy, but rather its delivery through locally feasible operational modifications. Vincristine was administered in routine care as a fixed 2 mg dose to simplify administration and reduce nursing complexity. Pegaspargase delivery was maintained through public procurement in order to improve continuity of access to a critical drug class. Treatment delivery was further organized around available monitoring and supportive-care capacity, rather than around the full resource assumptions of trial settings. Where clinically stable, parts of treatment were managed in the outpatient chemotherapy bay, while inpatient admission was prioritized for treatment initiation and management of complications. This framing is more accurate than describing daunorubicin 25 mg/m^2^ or pegaspargase use as departures from CALGB 10403, because those are already features of published CALGB 10403-based induction.

Supportive care and monitoring were likewise adapted to what could be delivered reliably in routine practice. During induction, patients underwent scheduled reassessment with complete blood counts, liver and renal function tests, coagulation profiles, blood glucose, pancreatic enzymes, and infection markers, while thrombosis imaging was obtained when clinically indicated. Supportive care within the routine pathway included infection prophylaxis, transfusion support, tumor lysis syndrome management, and escalation to inpatient care whenever clinically required. Accordingly, the major unresolved constraints in this public-sector setting were not the basic administration of induction chemotherapy itself, but limited access to advanced diagnostics, fragility in procurement continuity for critical agents, and restricted supportive-care and monitoring infrastructure. These were the system-level vulnerabilities most likely to translate into avoidable toxicity, treatment interruption, abandonment, or early mortality. No claim of equivalence to the full-intensity trial environment of CALGB 10403 is made [11,12,13,30]. The full input–capacity mapping for each component of the induction protocol is summarized in Supplementary Table S1.

### 2.8. Induction Therapy

All participants received the adapted induction regimen over 28 days as follows:

- Daunorubicin 25 mg/m^2^ intravenously on days 1, 8, 15, and 22
- Vincristine 2 mg (fixed dose) intravenously on days 1, 8, 15, and 22
- Prednisone 60 mg/m^2^ orally on days 1–21
- Pegaspargase 2500 U/m^2^ intramuscularly or intravenously on day 4
- Intrathecal cytarabine 70 mg on day 1, and intrathecal methotrexate 12 mg on days 8 and 15 for CNS prophylaxis

Extended induction was offered to patients with M2 marrow (5–25% blasts) on day 30 or when the day-30 assessment was delayed, consisting of weekly vincristine plus prednisone for 14 days, based on feasibility-oriented adaptations from comparable low-resource settings [11,12,13]. Cytarabine and methotrexate are generic drugs. Supportive care, including infection prophylaxis, transfusion support, and tumor lysis syndrome management, was delivered according to institutional protocols and was treated as an essential co-intervention for safe induction delivery, given the constrained ICU access and blood product availability in this setting.

### 2.9. Outcome Measures

The primary outcome was complete remission (CR) after induction (with or without extended induction), defined as bone marrow blasts of ≤ 5% with a neutrophil count of ≥ 1 × 10^9^/L and platelet count of ≥ 100 × 10^9^/L, and no extramedullary disease.

Secondary outcomes were: (1) early mortality, defined as death during induction or before assessment of induction response; (2) treatment abandonment, defined as failure to complete induction for non-medical reasons; (3) grade 3–4 toxicities, classified according to CTCAE version 5.0; (4) feasibility indicators, including the proportion of patients completing planned diagnostic workup (morphology, immunophenotyping) and induction therapy, operationalized as completion of intended pathway steps such as testing obtained, induction completed, and day-30 assessment performed to reflect service deliverability; and (5) economic feasibility, assessed by estimating the cost of diagnostic testing and induction chemotherapy relative to the mean monthly household income in Pakistan (PKR 82,000, approximately USD 290). Costs were assessed from the patient/household affordability perspective, reflecting the dominant out-of-pocket financing structure for diagnostics in this setting. Long-term endpoints, such as event-free and overall survival, were not assessed, given the induction-phase follow-up window of this study.

### 2.10. Data Collection

Demographic and clinical data were extracted from the medical records. Bone marrow aspirates were evaluated on day 30 and classified as complete remission (CR), partial remission (M2), or resistant disease (RD). Toxicities were recorded throughout induction and categorized according to the CTCAE grade. For feasibility outcomes, records were reviewed to determine whether key pathway steps were completed, immunophenotyping was performed, BCR-ABL1 results were recorded, day-30 assessments were documented, and induction was completed.

Patient-incurred out-of-pocket costs were extracted from hospital billing records and patient receipts, capturing charges for BCR-ABL1 RT-PCR, immunophenotyping by flow cytometry, bone marrow aspiration and biopsy procedures, and inpatient admission fees during induction. For the counterfactual analysis, the cost of a guideline-complete diagnostic package, including conventional cytogenetics (karyotype ± FISH), MRD testing, and Ph-like profiling, was estimated using PIMS finance schedules and external laboratory quotations.

### 2.11. Statistical Analysis

Descriptive statistics (means ± standard deviation for continuous variables and counts and percentages for categorical variables) were used to summarize the baseline characteristics and outcomes. Complete remission rates, early mortality, and toxicity frequencies were calculated with 95% confidence intervals (CIs). Given the implementation and feasibility aims, analyses focused on estimation with confidence intervals rather than hypothesis testing. Analyses were performed using IBM SPSS Statistics, Version 25.0 (IBM Corp., Armonk, NY, USA). No comparative hypothesis testing was pre-specified because the study was not powered for inferential comparison. Missingness was described for key variables, and analyses were performed using available case data without imputation.

### 2.12. Economic Assessment

Two scope limitations apply upfront: indirect household costs, including travel, lodging, and productivity loss, were not captured, and costs incurred after the induction phase were excluded. Affordability estimates should be interpreted within these limits.

Within these limits, a descriptive micro-costing comparison estimated (A) observed patient-incurred out-of-pocket expenditures and (B) the counterfactual cost of a guideline-complete diagnostic package. The analysis was conducted from both the patient (out-of-pocket payments) and public program (government-subsidized induction chemotherapy under the Sehat Card) perspectives. Resources used for observed costs were derived from itemized patient billing records and hospital finance schedules, and counterfactual diagnostic costs were obtained from institutional finance schedules and external laboratory quotations.

Affordability analysis compared these costs against national and quintile-specific income data from the Pakistan Bureau of Statistics Household Income and Expenditure Survey (PBS HIES 2024–25) [31], calculating the months of income required for observed and counterfactual costs across income brackets. Because hospitalization was managed through a hybrid inpatient-outpatient model, inpatient admission costs were calculated based on actual inpatient days rather than a fixed 28-day assumption. All costs are reported in FY2024–25 Pakistani Rupees (PKR) and converted to United States Dollars (USD) using the State Bank of Pakistan mid-year exchange rate (PKR 283.76 = USD 1) [32].

### 2.13. Ethical Considerations

The protocol was approved by the Institutional Ethics Review Committee of the Pakistan Institute of Medical Sciences (Reference: F-5-2/2024(ERRC)/PIMS). Study procedures were limited to data collection and analysis within routine care, and confidentiality safeguards were applied throughout.

## 3. Results

### 3.1. Patient Characteristics

**Figure 1:**
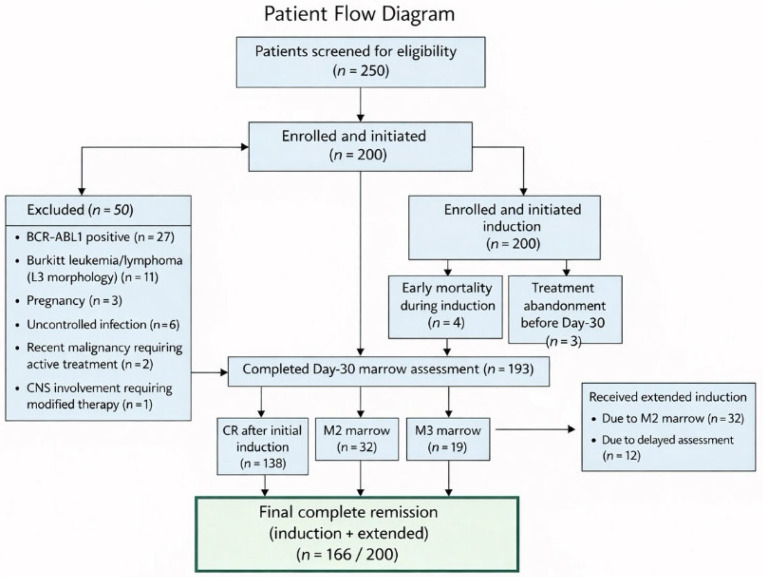
Patient flow and induction outcomes for adults with newly diagnosed Ph (−) ALL (December 2024–June 2025). CR: complete remission; M2: marrow (5–25% blasts); M3: marrow (>25% blasts).

Between December 2024 and June 2025, 200 adults with newly diagnosed Ph(−)ALL were prospectively enrolled (Figure 1).

The mean age was 30.30 ± 8.78 years, with 36.0% aged 18–25 years and 20.5% aged 36–50. Males comprised 65.5% of the cohort. A high disease burden at presentation was common, with 79 patients (39.5%) presenting with a white blood cell count ≥30 × 10^9^/L and 176 patients (88.0%) with platelet counts <50 × 10^3^/µL. Immunophenotyping identified B-ALL in 83 patients (41.5%) and T-ALL in 86 patients (43.0%) cases. Thirty-one patients (15.5%) were categorized as having Immunophenotype not classifiable (technical limitations). The baseline characteristics are presented in Table 1.

### 3.2. Induction Outcomes and Care Pathway Feasibility

Of the 200 enrolled patients, 193 (96.5%) were evaluable for the Day-30 bone marrow assessment; seven (3.5%) were not evaluable because four died during induction and three abandoned treatments before assessment. Baseline bone marrow morphology was obtained in all patients (100%). Immunophenotyping was performed in 169 of 200 patients (84.5%), while 31 (15.5%) were classified as “Immunophenotype not classifiable (technical limitations).” BCR-ABL1 RT-PCR was performed in all 200 patients, with results available in 100% of cases and a median turnaround time of 6 days (IQR: 4–8); 128 tests (64.0%) were performed in-house and 72 (36.0%) were outsourced.

Following initial induction, 138 of 193 evaluable patients achieved complete remission (CR), yielding an initial CR rate of 71.5%. On Day 30, 32 patients (16.6%) had M2 marrow and 19 (9.8%) had M3 marrow. Extended induction was administered to 44 patients (22.0%), 32 due to M2 marrow and 12 due to delayed assessment; 28 of these 44 patients (63.6%) achieved CR. The final CR rate, combining both induction phases, was 83.0% (166/200). Early induction-related mortality was 2.0% (4/200), and treatment abandonment was 1.5% (3/200).

Advanced diagnostic testing was not feasible in any enrolled patient. MRD testing by flow cytometry and Ph-like molecular profiling were not performed in any of the 200 patients (0%). These investigations are not covered under the Sehat Card program or any other provincial health scheme at this facility and remain unaffordable for the population served, with per-patient costs of USD 214 for MRD and USD 430 for Ph-like profiling, representing 2.6 and 5.2 months of income, respectively, for households in the poorest income quintile. A complete summary of outcomes and pathway completion indicators is presented in Table 2.

### 3.3. Treatment-Related Toxicity

Grade 3–4 toxicities during induction were captured primarily through scheduled outpatient follow-up visits, including hematologic, hepatic, renal, coagulation, metabolic, pancreatic, and infection-related monitoring; thrombosis was assessed with D-dimer testing and, when clinically indicated, Doppler ultrasonography or CT imaging.

Febrile neutropenia was documented in 30 patients (15.0%), hepatotoxicity in 20 (10.0%), and sepsis in 15 (7.5%), including culture-positive cases. Hypofibrinogenemia was identified in 25 patients (12.5%) and hyperglycemia requiring insulin in 13 (6.5%). Pancreatitis and thrombosis were documented in nine (4.5%) and five (2.5%) patients, respectively.

Active daily inpatient monitoring was not conducted as part of routine practice, and no systematic proxy measures, such as ICU transfers, vasopressor use, or transfusion volumes, were recorded in this cohort. Therefore, toxicity figures should be interpreted as detected toxicity and likely lower-bound estimates within an outpatient-dominant monitoring model in a resource-constrained setting. Toxicity data are summarized in Table 3.

### 3.4. Economic Feasibility and Affordability

To avoid misinterpretation, costs are reported separately as (A) observed patient-incurred out-of-pocket expenditure for tests performed and (B) a counterfactual guideline-complete diagnostic package representing investigations required for contemporary risk stratification but not performed in this cohort.

Induction chemotherapy delivery was largely subsidized through the Sehat Card program, with a government program cost of USD 1107 (PKR 314,127) per patient. In contrast, none of the advanced diagnostic investigations, conventional cytogenetics, MRD testing, or Ph-like profiling are covered under the Sehat Card program or any other available public subsidy at this facility. The counterfactual full diagnostic package (karyotype ± FISH, single-time-point MRD, and Ph-like profiling) was estimated to cost USD 929 (PKR 263,617) per patient. The ratio of this diagnostic add-on cost to the subsidized induction program cost was 0.84, indicating that including comprehensive risk stratification would increase the total publicly financed per-patient expenditure by approximately 84%.

Patients incurred modest observed out-of-pocket costs: BCR-ABL1 RT-PCR (approximately USD 79.3), immunophenotyping (approximately USD 5.29), bone marrow aspiration procedure fee (approximately USD 25), and inpatient admission fees based on actual inpatient days under the hybrid model (approximately USD 2 per night). The estimated typical observed total out-of-pocket expenditure was approximately PKR 33,850 (USD 119). The detailed cost components are presented in Table 4.

### 3.5. Affordability Analysis

Using the Pakistan Bureau of Statistics income data from the HIES 2024–25, the number of months of household income required for each cost scenario was calculated as follows: The observed OOP total (PKR 33,850, approximately USD 119) represents 0.81 months of income for households in the poorest income quintile (Q1) and 0.41 months for the national mean, a burden that, while not trivial, falls within a range potentially manageable for some households, given the Sehat Card subsidy for chemotherapy.

The counterfactual diagnostic add-on (PKR 263,617, approximately USD 929) represents 6.30 months of income for Q1 households and 3.21 months for the national mean figures, which place guideline-complete risk stratification financially beyond the reach of the majority of patients served by this facility. Adding one further MRD time-point (total PKR 324,343, approximately USD 1144) increases this to 7.75 months for Q1 households and 3.95 months for the national mean. These data are summarized in Table 5.

## 4. Discussion

### 4.1. Principal Findings and Contextualized Outcomes

This prospective cohort study of 200 adults with Philadelphia chromosome-negative ALL at a resource-restricted public hospital in Pakistan demonstrated the operational feasibility of a pragmatically adapted pediatric-inspired induction regimen. The observed complete remission (CR) rate of 83.0%, together with low early induction-related mortality (2.0%) and treatment abandonment (1.5%), is encouraging in this setting. These outcomes compare favorably with other LMIC reports, where CR rates often fall below 70% and induction-related mortality can exceed 20% [26,27,28,29]. However, these findings should be interpreted as signals of operational deliverability rather than evidence of equivalence to full-intensity protocols in high-income settings, where CR rates routinely exceed 90% [3,4,30]. The remaining gap likely reflects system-input deficits, particularly the exclusion of molecular diagnostics from coverage under the Sehat Sahulat Program [22,23].

### 4.2. Implementation Science and the Cancer Groundshot Approach

Successful delivery of a 28-day induction protocol within an 18-bed oncology ward reflects an adaptive response within routine public-sector oncology care. Rather than functioning as a fixed bottleneck, limited bed capacity was partly offset by a hybrid inpatient-outpatient model, similar to strategies used in other high-volume Pakistani centers transitioning to daycare-based induction [33]. This aligns with the “Cancer Groundshot” approach, which emphasizes implementation of high-value interventions through frugal innovation before pursuit of resource-intensive breakthroughs that the local infrastructure cannot yet sustain [34]. These findings suggest that service readiness depends not only on infrastructure, but also on adaptive workflows and care organization [35].

### 4.3. Healthcare Delivery Fit and Service Packaging

The high Day-30 evaluability (96.5%) is an important process-of-care indicator, reflecting effective care coordination and patient retention during intensive induction. This is especially relevant where long-term follow-up is often limited, making early process metrics useful proxies for program quality [9,15]. The low abandonment rate of 1.5%, compared with reports from other LMIC settings where financial toxicity and travel burden contribute substantially to default [8], suggests that subsidized chemotherapy is most effective when delivered as part of a broader “service package” that includes diagnostic access, skilled nursing coverage, infection control, and escalation pathways [22,23]. The use of extended induction in 22% of patients further illustrates adaptive clinical capacity in the absence of formal re-induction protocols or transplantation access [11,12,13].

### 4.4. Diagnostic Capacity, Biological Risk Stratification, and the Ph-Testing Gap

The finding that 15.5% of cases were categorized as “Immunophenotype not classifiable (technical limitations)” due to incomplete antibody panels, including absence of cCD79a, cCD3, and MPO, or sample degradation, mirrors diagnostic constraints reported across LMIC oncology programs [25,26]. This limits accurate risk stratification, which remains central to contemporary ALL management [36]. Without routine cytogenetics, FISH, or molecular profiling, clinicians cannot identify high-risk lesions such as BCR-ABL1 rearrangements, Ph-like ALL, or other kinase-activating abnormalities [7,24]. The absence of flow cytometry-based MRD testing further restricts treatment tailoring and response-guided intensification or de-escalation [6].

A related gap is the Ph-testing asymmetry. Although BCR-ABL1 RT-PCR was performed in all patients, the median 6-day turnaround for outsourced testing creates a “wait-to-treat” paradox in which induction begins before Ph status is confirmed, potentially delaying tyrosine kinase inhibitor initiation in Ph-positive cases [7,24]. This delay reflects financing and infrastructure limitations, not merely laboratory throughput, because the current Sehat Card structure subsidizes treatment delivery but not the diagnostic infrastructure needed for precision-based treatment assignment [22,23].

MRD testing and Ph-like molecular profiling were not performed in any enrolled patient. At this facility, neither is covered under the Sehat Sahulat Program, and both remain available only at select private or research centers at costs equivalent to 2.6 and 5.2 months of income, respectively, for households in the poorest income quintile. This enforces a one-size-fits-all treatment model and perpetuates biological inequity. A practical policy step would be staged inclusion of a minimum subsidized diagnostic panel, particularly cytogenetics and MRD where feasible, linked explicitly to treatment decision pathways under an expanded Sehat Card oncology package [22,23].

### 4.5. Treatment Implementation Under Pharmacologic Constraints

Asparaginase, a core component of pediatric-inspired induction, presents recurrent supply and quality challenges in LMIC procurement environments [18,19]. At the same time, host susceptibility contributes to toxicity; class II HLA variation has been associated with pegaspargase hypersensitivity, indicating that immune-mediated reactions should not be conflated with product quality or supply failures [37]. Reports of substandard formulations, including regulatory alerts relevant to Pakistan, indicate that procurement and formulation quality are patient-safety issues upstream of the clinical encounter [19,38]. Although pegaspargase was secured through public procurement for this cohort without reported supply interruption, this cannot be assumed for future cycles or other centers in the region [18,19,38]. Physicochemical quality variation across manufacturers further supports procurement quality assurance as a direct determinant of clinical safety [38]. Until supply stability and robust quality assurance are established, simplified stepped-intensity regimens such as those used in Rwanda and Cambodia remain reasonable adaptive strategies [11,13].

The observed grade 3–4 toxicities, including febrile neutropenia (15.0%), sepsis (7.5%), hepatotoxicity (10.0%), and hypofibrinogenemia (12.5%), were within the expected range for this regimen but should be interpreted as detected toxicity rather than absolute burden. In resource-limited settings where malnutrition is prevalent, altered chemotherapy pharmacokinetics may amplify toxicity even at reduced doses [39]. Because active daily inpatient monitoring was not routine, and no systematic proxy measures such as ICU transfer rates, vasopressor use, or transfusion volumes were recorded, these figures are likely lower-bound estimates. Future studies should incorporate such proxy indicators to provide more complete safety estimates [35].

### 4.6. Patient Safety, Early Quality Indicators, and the ICU Capacity Constraint

The low induction-related mortality (2.0%) aligns with reports from successful LMIC programs in Rwanda and Latin America, suggesting that basic safety standards can be achieved in this operating environment [13,28]. Structured use of extended induction also reflects adaptive clinical capacity in the absence of more advanced options such as transplantation [11,12]. However, severe complications remain difficult to rescue once escalation is required, given Pakistan’s limited ICU capacity of approximately two beds per 100,000 population [20]. Embedding standardized sepsis recognition pathways, including explicit fever, hypotension, and bleeding thresholds, could convert best-effort care into more reproducible safety processes. This is particularly relevant because stewardship programs in Pakistani ICUs have already shown reductions in mortality and antibiotic costs [40].

### 4.7. Treatment Abandonment and the Absence of Default Tracking

The low abandonment rate should be interpreted alongside an important structural limitation: PIMS does not currently have infrastructure for personalized follow-up of patients who default. In the absence of a unified national cancer registry and under severe staffing constraints, there is no practical capacity for outreach or community-based tracking [41]. Once treatment is abandoned, patients are effectively lost to the system. This is therefore a weakness of program maintenance rather than of the regimen itself, and it is not addressed by the inpatient-focused design of the Sehat Card, which covers treatment initiation but not retention, transport, or outreach support [22,23].

### 4.8. Financial Protection, Affordability, and the Diagnostic-Therapeutic Asymmetry

The economic analysis documents a clear asymmetry: induction chemotherapy is largely financed through the Sehat Sahulat Program at a program cost of USD 1107 per patient [22,23], whereas the counterfactual guideline-complete diagnostic package costs USD 929 per patient and is not covered under any public subsidy mechanism at this facility. For households in the poorest income quintile, the diagnostic add-on alone represents 6.3 months of income, placing biology-driven care beyond reach for most patients served by this center. Indirect costs, including travel, lodging, and productivity loss, were not captured and likely add to this burden.

This pattern is not unique to Pakistan and reflects broader fragmentation of cancer financing and timely access across LMIC settings [34,42,43]. The most defensible near-term response is staged diagnostic coverage within the Sehat Card architecture, beginning with a minimum panel tied to explicit treatment decisions and expanding as laboratory capacity and public financing improve [22,23].

### 4.9. Health Equity, Access, and the Intersectionality of Late Presentation

Geographic and socioeconomic barriers shape ALL outcomes before patients reach treatment facilities, and these constraints often intersect [44]. The geographic profile of this cohort reflects the concentration of tertiary oncology services in a limited number of urban centers [14,43]. The high disease burden at presentation, including elevated WBC counts and severe thrombocytopenia, resembles other LMIC cohorts [26,27] and likely reflects delayed pathways to care driven by travel burden, limited health literacy, and delayed engagement with formal health services [15,16,42]. These are access-friction problems rather than biological ones and require both supply-side and demand-side responses. Pakistan’s lack of a unified national cancer registry further limits incidence estimation and rational planning [14,41]. A networked hospital-based registry using harmonized minimum datasets would offer a practical near-term step, while tele-hematology and subsidized transport could help reduce geographic access barriers.

### 4.10. Policy and Governance Implications

Improving adult ALL outcomes in Pakistan will require policy reform beyond chemotherapy selection alone. Priority actions include incorporation of cytogenetics, MRD testing, and molecular profiling into essential benefit packages [10,17]; stronger procurement and quality-assurance mechanisms for agents such as asparaginase [18,19,38]; expansion of ICU capacity, transfusion services, and oncology nursing [20]; development of registry-linked data systems for longitudinal outcomes [41]; prospective planning for targeted therapies and transplantation pathways [7,24]; and regional collaboration in procurement, training, and research across South Asia [34,44]. Overall, the feasibility of a regimen is inseparable from the governance of diagnostics, supportive care, and procurement systems.

### 4.11. Strengths and Limitations

This study has several strengths, including prospective consecutive enrollment, explicit feasibility endpoints, integration of economic context with clinical outcomes, and use of a counterfactual cost model that makes structural financing gaps visible. The high Day-30 evaluability also strengthens confidence in ascertainment of early induction outcomes.

Important limitations should also be acknowledged. This was a single-center study in a public-sector setting, which may limit generalizability to other hospitals with different leadership, workflows, or procurement conditions. The cohort was relatively young and fit, which limits extension of these findings to older or frailer adults with ALL. The absence of a comparator arm prevents conclusions about superiority or equivalence. Diagnostic limitations, including total absence of cytogenetics, MRD testing, and Ph-like profiling, reduce biological interpretability and preclude subgroup analyses by disease risk.

We also did not perform inferential comparisons across lineage or income strata. This reflects structural rather than analytic constraints, because all patients lacked access to advanced diagnostics and the 15.5% unclassifiable immunophenotype rate further limited lineage-specific interpretation. Toxicity may have been underestimated because ascertainment relied on scheduled outpatient monitoring without systematic inpatient proxy measures. Finally, the economic analysis did not include indirect household costs such as travel, lodging, or productivity loss.

### 4.12. Research Agenda for Implementation-Focused Oncology

The next logical step is a multicenter prospective implementation study evaluating whether this model can be scaled across public hospitals with different staffing, diagnostic, and procurement capacity. Such work should assess the impact of a minimum subsidized diagnostic panel on treatment decisions, examine interventions to improve asparaginase procurement stability and quality assurance, and incorporate cost-effectiveness analyses that include both direct and indirect patient costs under the Sehat Card framework [22,23]. Longer-term agendas should also include survivorship-focused follow-up. Evidence from limited-resource childhood cancer settings underscores that equitable cancer care cannot stop at remission but must also address late effects, continuity of care, and preventable mortality after initial treatment [45]. Progress from isolated feasibility studies toward multicenter implementation evidence and stronger trial infrastructure will be necessary for sustainable improvement in ALL outcomes in resource-constrained settings [46].

## 5. Conclusions

A pragmatically adapted, pediatric-inspired induction regimen was operationally deliverable in a resource-restricted public tertiary hospital and was associated with an 83.0% complete remission rate, low induction-related mortality, and low treatment abandonment. These findings support the feasibility of adapted pediatric-inspired induction in this setting but not equivalence to full-intensity protocols. Closing the remaining outcome gap will likely depend less on further regimen intensification than on strengthening diagnostic access, procurement reliability, and early supportive-care systems.

## Figures and Tables

**Table 1:** Baseline Patient Characteristics (*N* = 200).

Characteristic	Value
Age, mean ± SD (years)	30.30 ± 8.78
18–25 years, *n* (%)	72 (36.0)
26–35 years, *n* (%)	87 (43.5)
36–50 years, *n* (%)	41 (20.5)
Gender	
Male, *n* (%)	131 (65.5)
Female, *n* (%)	69 (34.5)
ECOG PS 0–1, *n* (%)	147 (73.5)
WBC ≥ 30 × 10^9^/L, *n* (%)	79 (39.5)
Platelets < 50 × 10^3^/µL, *n* (%)	176 (88.0)
Geographic origin, *n* (%)	
Punjab	135 (67.5)
Khyber Pakhtunkhwa	45 (22.5)
Baluchistan	12 (6.0)
Sindh	8 (4.0)
Immunophenotype, *n* (%)	
B-ALL	83 (41.5)
T-ALL	86 (43.0)
Immunophenotype not classifiable (technical limitations)	31 (15.5)
Prior corticosteroid exposure, *n* (%)	23 (11.5)

Footnotes: Percentages are calculated as (*n*/*N*) × 100 using the denominator shown in the *n*/*N* column and rounded to 1 decimal place. ALL = acute lymphoblastic leukemia; ECOG = Eastern Cooperative Oncology Group; SD = standard deviation; WBC = white blood cell count.

**Table 2:** Induction Outcomes and Care Pathway Completion Indicators (*N* = 200).

Outcome/Pathway Step	*N*	%
Diagnostic pathway completion		
Bone marrow morphology obtained	200/200	100.0
Immunophenotyping obtained and resulted	169/200	84.5
BCR-ABL1 RT-PCR obtained	200/200	100.0
BCR-ABL1 result available in clinical record	200/200	100.0
BCR-ABL1 performed in-house	128/200	64.0
BCR-ABL1 outsourced	72/200	36.0
Median BCR-ABL1 turnaround time	6 days (IQR 4–8)	—
MRD testing (flow cytometry) performed	0/200	0.0
Ph-like molecular profiling performed	0/200	0.0
Treatment pathway completion		
Day-30 marrow assessment performed	193/200	96.5
Induction completed	193/200	96.5
Extended induction delivered	44/200	22.0
— Due to M2 marrow at Day-30	32/44	72.7
— Due to delayed Day-30 assessment	12/44	27.3
Clinical outcomes		
Initial CR after first induction	138/193	71.5
M2 marrow at Day-30	32/193	16.6
M3 marrow at Day-30	19/193	9.8
CR after extended induction	28/44	63.6
Final CR (induction ± extended induction)	166/200	83.0
Early induction-related mortality	4/200	2.0
Treatment abandonment	3/200	1.5

Footnotes: Percentages were calculated as (*n*/*N*) × 100 using the denominator shown and rounded to one decimal place. CR, complete remission; IQR, interquartile range; MRD, measurable residual disease. MRD and Ph-like profiling were unavailable at this facility and excluded from Sehat Card coverage.

**Table 3:** Grade 3–4 Toxicities during Induction (*N* = 200).

Toxicity	*N*	%
Febrile neutropenia	30	15.0
Sepsis	15	7.5
Hepatotoxicity	20	10.0
Hypofibrinogenemia	25	12.5
Hyperglycemia requiring insulin	13	6.5
Pancreatitis	9	4.5
Thrombosis	5	2.5

Footnotes: Percentages were calculated using *N* = 200. Toxicity categories are not mutually exclusive; a patient may contribute to more than one category of toxicity. Figures represent the detected toxicity based on outpatient monitoring visits; systematic inpatient proxy measures were not collected.

**Table 4:** Economic Feasibility: Observed Patient-Incurred Costs Versus Counterfactual Guideline-Complete Diagnostic Package (FY2024–25).

Item	PKR (FY2024–25)	USD	Source	Status
Panel A: Observed patient-incurred costs				
BCR-ABL1 RT-PCR (t(9;22) molecular)	22,500	79.3	Patient billing records	Captured (observed OOP)
Immunophenotyping (flow cytometry)	1500	5.29	Patient billing records	Performed (partial OOP)
Bone marrow aspiration/biopsy procedure	7000	25.0	Patient billing records	Performed (partial OOP)
Inpatient admission (per night)	500 per night	2.0 per night	Hospital fee schedule	Subsidized; small OOP; calculated on actual inpatient days
Estimated observed OOP total	~33,850	~119		
Panel B: Counterfactual diagnostics (not performed)				
Conventional cytogenetics (karyotype ± FISH)	80,873	285	Finance schedule/external quote	Not covered under Sehat Card; not available routinely
MRD testing (flow cytometry, single time-point)	60,726	214	Finance schedule/external quote	Not covered under Sehat Card; not available routinely
Ph-like ALL profiling panel	122,019	430	External laboratory quote	Not covered under Sehat Card; not available routinely
Total counterfactual add-on	263,617	929	Sum of above	Hypothetical additional cost
Panel C: Program cost				
Induction chemotherapy (PIMS protocol)	314,127	1107	Hospital pharmacy/finance	Covered under Sehat Card program

Footnotes: All costs are reported in FY2024–25 PKR and converted to USD using the State Bank of Pakistan mid-year exchange rate (PKR 283.76 = USD 1). The observed total OOP represents an estimated typical per-patient expenditure derived from itemized billing components; inpatient costs were calculated from actual inpatient days, not a fixed 28-day assumption. Indirect household costs (travel, lodging, productivity loss) are not included and represent an additional, unquantified burden. Sehat Card coverage applies to induction chemotherapy (Panel C) but does not extend to any item in Panel B.

**Table 5:** Affordability Analysis: Required Months of Household Income for Observed and Counterfactual Cost Scenarios (FY2024–25).

Income Level	Monthly Income (PKR)	Months for Observed OOP (PKR 33,850)	Months for Counterfactual Add-On (PKR 263,617)	Months for Add-On + 1 Additional MRD (PKR 324,343)
Poorest quintile (Q1 mean)	41,851	0.81	6.30	7.75
National mean	82,179	0.41	3.21	3.95

Footnotes: Months of income were calculated as cost ÷ monthly income. For example, 33,850 ÷ 41,851 = 0.81. Income data were sourced from the PBS HIES 2024–25. Exchange rate: PKR 283.76 = USD 1 (State Bank of Pakistan mid-year rate, FY2024–25). The counterfactual add-on reflects the cost of conventional cytogenetics (karyotype ± FISH), single-time-point MRD testing, and Ph-like profiling, none of which are covered under the Sehat Card program.

## Data Availability

The datasets analyzed are available at Figshare: https://doi.org/10.6084/m9.figshare.31412657.

## Supplementary Materials

The following supporting information can be downloaded at: https://www.mdpi.com/article/10.3390/healthcare14081038/s1, Supplementary Table S1: Input–capacity matrix for delivering pediatric-inspired induction for adult Ph (−) ALL in a Pakistani public-sector tertiary oncology setting.

## Abbreviations

ALL	acute lymphoblastic leukemia
BCR-ABL1	breakpoint cluster region-Abelson 1 fusion gene
CR	complete remission
CTCAE	Common Terminology Criteria for Adverse Events
ECOG	Eastern Cooperative Oncology Group
LMIC	low- and middle-income country
MRD	measurable residual disease
PIMS	Pakistan Institute of Medical Sciences
RT-PCR	reverse transcription polymerase chain reaction
SD	standard deviation
WBC	white blood cell
OOP	Out of pocket
