# Supplementary material for: Feasibility and Policy Implications of a Pragmatically Adapted Pediatric-Inspired Induction Regimen for Adults with Acute Lymphoblastic Leukemia in a Resource-Restricted Setting: A Prospective Observational Study
Source: Healthcare (Basel). 2026 Apr 14;14(8):1038. doi: 10.3390/healthcare14081038 (PMC13116484; doi:10.3390/healthcare14081038)
Supplement: Supplementary file 1 [file healthcare-14-01038-s001.zip › healthcare-4198135-supplementary.pdf]

Feasibility and Policy Implications of a Pragmatically Adapted Pediatric-Inspired Induction Regimen for Adults with Acute Lymphoblastic Leukemia in a Resource-Restricted Setting: A Prospective Observational Study

Supplementary Table S1. Input–capacity matrix for delivering pediatric-inspired induction for adult Ph(–) ALL in a Pakistani public-sector tertiary oncology setting

Purpose: Describes service inputs required for safe, protocol-concordant induction and how common public-sector constraints may affect feasibility endpoints (diagnostic feasibility, therapeutic feasibility, early outcomes, and economic feasibility). This is context for interpretation, not a regimen comparison.

Service input domain	Benchmark / ideal for safe induction	Pakistan public-sector availability (typical tertiary setting)	Feasibility implications (what it may drive)
**Baseline diagnostics** (morphology + immunophenotyping)	Rapid diagnostic confirmation with standardized immunophenotyping for lineage classification	Generally available at tertiary centers; turnaround time and completeness may be variable	Delayed start of appropriate pathway; incomplete classification may limit protocol matching
**Cytogenetics / FISH**	Karyotyping + FISH available for all eligible patients with actionable turnaround	Available at select urban labs/centers; frequently delayed and/or unaffordable; not consistently accessible across patients	Lower *diagnostic feasibility*; risk stratification constrained; may increase empiric decisions and non-receipt of testing
**Molecular testing (PCR panels beyond BCR-ABL)**	Broad PCR/targeted panels to identify key lesions (e.g., Ph-like surrogates where relevant)	Limited and center-dependent; often restricted to BCR-ABL PCR where available; broader profiling uncommon	Missed actionable subtypes; limits risk-adapted intensification/de-intensification; may affect early outcomes via

			suboptimal matching
**MRD monitoring (flow and/or PCR)**	MRD measured at defined timepoints with QA and clinical pathways linked to results	Rare outside research; not routinely available for standard public care	Lower *diagnostic feasibility*; prevents MRD-guided decisions; complicates benchmarking against HIC outcomes
**Asparaginase access + quality assurance**	Consistent supply of quality-assured formulations; standardized toxicity monitoring; cold-chain integrity	Supply interruptions occur; formulation/quality assurance may be variable; cold-chain reliability may be challenging in some settings	Interruptions/dose omissions (lower *therapeutic feasibility*); higher toxicity risk if monitoring inconsistent; may raise early mortality/toxicity
**Drug procurement reliability (core cytotoxics, supportive meds)**	Predictable procurement; minimal stock-outs; standardized protocols	Periodic stock-outs and delays reported; supportive meds may be intermittently unavailable	Treatment interruptions, protocol deviations; potentially higher toxicity or relapse risk; documentation gaps common
**Transfusion support (RBCs/platelets/FFP/cryoprecipitate)**	Reliable component availability with rapid turnaround and screening	Shortages common; platelet and cryoprecipitate constraints can be prominent; reliance on replacement donation is frequent	Bleeding risk during induction; delays in chemotherapy due to cytopenia support gaps; higher grade 3–4 toxicity and early mortality risk

**Infection prevention + control**	Adequate isolation capacity, nurse staffing, and IPC supplies; timely cultures and antibiotics	Overcrowding and limited isolation space can constrain IPC; staffing ratios may limit close monitoring	Higher risk of sepsis; delayed recognition and management; may increase grade 3–4 toxicity and early mortality
**Antimicrobial access (broad-spectrum + antifungals)**	Protocolized prophylaxis/empiric coverage; steady access to key agents	Access variable; shortages/cost barriers can limit timely therapy or optimal agents	Higher infection complications; longer interruptions; higher ICU escalation and early mortality risk
**Critical care escalation (ICU/HDU beds, ventilatory support)**	Clear ICU pathways; sufficient beds; rapid escalation for septic shock/bleeding/organ failure	ICU capacity constrained; delays in escalation may occur due to bed scarcity	Higher early mortality; may distort toxicity profile (events occur but care not available)
**Oncology nursing + pharmacy support**	Oncology-trained nursing; chemo verification processes; clinical pharmacy involvement	Workforce shortages; limited oncology-trained nurses/pharmacists in many public settings	Medication delays/errors risk; reduced monitoring intensity; affects feasibility and safety outcomes
**Laboratory monitoring capacity** (CBC, chemistries, coagulation, LFTs)	High-frequency monitoring during induction with rapid turnaround	Basic labs typically available; turnaround and frequency may be constrained by volume/workflow and cost	Delayed detection of complications (TLS, hepatotoxicity, coagulopathy); may increase severe toxicity
**Patient financial protection**	Diagnostics and drugs covered; minimal catastrophic expenditure;	Substantial out-of-pocket costs persist; ancillary costs (travel, lodging, lost	Lower *economic feasibility*; higher non-receipt of tests,

	travel/lodging support	wages) often high	interruptions, and abandonment
**Care navigation / follow-up infrastructure**	Structured follow-up, adherence support, and rapid re-admission pathways	Fragmented follow-up is common; travel distance and costs limit continuity	Higher abandonment and unplanned interruptions; under-ascertainment of outcomes if follow-up incomplete

Abbreviations: ALL, acute lymphoblastic leukemia; Ph(–), Philadelphia chromosome–negative; FISH, fluorescence in situ hybridization; PCR, polymerase chain reaction; MRD, minimal residual disease; QA, quality assurance; ICU, intensive care unit; HDU, high-dependency unit; IPC, infection prevention and control; CBC, complete blood count; LFTs, liver fu
